# Assessment of Beta-2 Microglobulin Gene Edited Airway Epithelial Stem Cells as a treatment for Sulfur Mustard Inhalation

**DOI:** 10.3389/fgeed.2022.781531

**Published:** 2022-02-07

**Authors:** Meisam Naeimi Kararoudi, Alfahdah Alsudayri, Cynthia L. Hill, Ezgi Elmas, Yasemin Sezgin, Aarohi Thakkar, Mark E. Hester, Daniel T. Malleske, Dean A. Lee, Matthew L. Neal, Mark R. Perry, Jill A. Harvilchuck, Susan D. Reynolds

**Affiliations:** ^1^ Nationwide Children’s Hospital, Columbus, OH, United States; ^2^ Molecular, Cellular, and Developmental Biology Graduate Program, The Ohio State University, Columbus, OH, United States; ^3^ Battelle Memorial Institute, Columbus, OH, United States

**Keywords:** Cas9, ribonucleoprotein complex, tracheobronchial epithelial tissue specific stem cell, sulfur mustard, inhalation, medical countermeasure

## Abstract

Respiratory system damage is the primary cause of mortality in individuals who are exposed to vesicating agents including sulfur mustard (SM). Despite these devastating health complications, there are no fielded therapeutics that are specific for such injuries. Previous studies reported that SM inhalation depleted the tracheobronchial airway epithelial stem cell (TSC) pool and supported the hypothesis, TSC replacement will restore airway epithelial integrity and improve health outcomes for SM-exposed individuals. TSC express Major Histocompatibility Complex (MHC-I) transplantation antigens which increases the chance that allogeneic TSC will be rejected by the patient’s immune system. However, previous studies reported that Beta-2 microglobulin (B2M) knockout cells lacked cell surface MHC-I and suggested that B2M knockout TSC would be tolerated as an allogeneic graft. This study used a Cas9 ribonucleoprotein (RNP) to generate B2M-knockout TSC, which are termed Universal Donor Stem Cells (UDSC). Whole genome sequencing identified few off-target modifications and demonstrated the specificity of the RNP approach. Functional assays demonstrated that UDSC retained their ability to self-renew and undergo multilineage differentiation. A preclinical model of SM inhalation was used to test UDSC efficacy and identify any treatment-associated adverse events. Adult male Sprague-Dawley rats were administered an inhaled dose of 0.8 mg/kg SM vapor which is the inhaled LD_50_ on day 28 post-challenge. On recovery day 2, vehicle or allogeneic Fisher rat UDSC were delivered intravenously (*n* = 30/group). Clinical parameters were recorded daily, and planned euthanasia occurred on post-challenge days 7, 14, and 28. The vehicle and UDSC treatment groups exhibited similar outcomes including survival and a lack of adverse events. These studies establish a baseline which can be used to further develop UDSC as a treatment for SM-induced airway disease.

## Introduction

A tissue specific stem cell is defined as a cell that replaces itself and acts as a progenitor for each of the differentiated cell types that are found in the stem cell’s home tissue. The tracheobronchial epithelial tissue specific stem cell (TSC) regenerates the pseudostratified airway epithelium and resides in the tracheobronchial regions of the mouse, rat, and human respiratory tract. Clonal analysis in human (Engelhardt et al., 1995; White et al., 2016) and lineage-tracing in mice (Hong et al., 2003; Hong et al., 2004; Rock et al., 2009; Ghosh et al., 2011a) indicated that the TSC was a basal cell subtype which generated basal, ciliated, secretory epithelial cells, and a variety of rare epithelial cell types. The TSC’s ability to self-renew and regenerate all airway epithelial cell types suggests that it could be used as a treatment for acute or chronic diseases that compromise conducting airway epithelial function.

Sulfur Mustard (SM) is a vesicating chemical warfare agent that is relatively easy to synthesize, stockpile, and deploy. Although SM use is prohibited, SM exposures continue to occur and result in sustained eye, skin, and lung injuries (reviewed in White et al. (2016)). Conducting airway epithelial damage accounts for most of the mortality and morbidity after SM inhalation. This pathology includes plastic bronchitis, an obstructive condition in which rubbery/chalk-like plugs (casts) occlude the airway lumen and impede gas exchange (White et al., 2016), airway epithelial necrosis (Perry et al., 2021), and depletion of the basal cell pool (Ghanei et al., 2011). Persistent epithelial pathology, including regions of epithelial denudation, indicates that SM exposure causes an aberrant epithelial repair process that lasts months to years. Further, the high rate of pulmonary infection in SM exposure survivors suggests that abnormal repair compromises mucociliary clearance, an essential epithelial function (White et al., 2016). Collectively, these data indicate that TSC depletion underlies the SM-induced airway epithelial repair defect.

A medical countermeasure (MCM, (Smith, 2009; Keyser et al., 2014)) for SM inhalation which is safe and decreases SM-associated morbidity in rats by 50% is likely to undergo further development. For example, preclinical studies demonstrated that intratracheal instillation of Tissue Plasminogen Activator (tPA) had a high safety profile, that SM-induced casts were cleared, and that this treatment decreased SM-associated morbidity (Veress et al., 2015). Since these data suggested that the acute effects of SM inhalation could be mitigated, tPA was transitioned to a National Institutes of Health (NIH)-Biomedical Advanced Research and Development Authority (BARDA) product. However, use of the tPA MCM requires advanced medical skills and is most effective when it is delivered within hours of exposure. The chaotic nature of a mass SM exposure event and the narrow treatment window for tPA indicates a need for medications that prevent or reverse SM-induced chronic lung disease. An American Thoracic Society (ATS) Workshop report suggested that TSC therapy was a promising adjunct to existing MCM (Summerhill et al., 2017).

TSC therapy has not been developed for any lung disease; however, several approaches have been considered. Autologous stem cell therapy has been prioritized because it would not cause immune system-mediated rejection. However, enthusiasm for this approach is tempered by data indicating that the TSC is damaged by SM inhalation and that TSC are aged/exhausted in chronic lung disease (Ghanei et al., 2011; McGraw et al., 2017). Considering these issues, an effective autologous stem cell therapy would likely require prospective collection, amplification, and storage of TSC. While our published studies demonstrate that TSC biobanking is feasible (Hayes et al., 2018), the costs and logistical concerns could be substantial. An alternative form of autologous stem cell therapy would use TSC that are generated from patient-derived induced pluripotent stem cells (iPSC). This approach has several drawbacks: 1) the time needed to generate iPSC; 2) the embryonic phenotype of iPSC-derived basal cells; and 3) abnormal expression of genes that promote rejection (reviewed in Chiou et al. (2016)). A third type of therapy would employ allogeneic TSC. This approach would require immuno-suppression and increase the already high risk of infection in SM-exposed individuals and chronic lung disease patients. Collectively, these limitations indicate a need for an innovative approach to TSC therapy.

The immune system uses several mechanisms to identify damaged or foreign cells. Class 1 Major Histocompatibility Antigen (MHC-I) proteins are the primary activators of immune rejection. In contrast, AB-Blood Group Antigens (BGA) are minor contributors to the rejection process. We reasoned that MHC-I^negative^, O-BGA TSC would avoid immune cell detection and function as Universal Donor Stem Cells (UDSC). MHC-I proteins are highly polymorphic, and it is estimated that there are ∼1 billion MHC-I genetic variants within the human population (Wang et al., 2015). This diversity makes it unlikely that a perfectly matched TSC donor would be available and indicates that a CRISPR/Cas9 (Cas9)-mediated MHC-I knockout (KO) would require a donor specific approach. In contrast with MHC-I, Beta-2 microglobulin (B2M) is highly conserved. Previous work demonstrated that the MHC-I proteins are non-covalently associated with B2M and expression of MHC-I on the cell surface is dependent on co-expression of B2M. A previous study reported that Cas9 mediated B2M KO in iPSC resulted in an MHC-I null phenotype (Wang et al., 2015).

A small number of studies reported Cas9 mediated gene editing of TSC and suggested that a similar strategy could be used to generate B2M-KO-TSC (Krishnamurthy et al., 2019). Although the most common approach to gene editing primary cells uses viral vectors to deliver Cas9 and the guide RNA (gRNA), many off target edits were reported. In contrast, use of a Cas9 ribonucleoprotein (RNP) and electroporation delivers a high concentration of Cas9 which is short lived and results in fewer non-specific gene edits (Everman et al., 2018). Since we previously reported that electroporation of Cas9/RNP has low off-target effects in primary human cells (Naeimi Kararoudi et al., 2020), this study delivered a Cas9 chimeric protein and pre-transcribed gRNA to TSC via electroporation.

We previously used the naphthalene (NA) chemical injury model to determine if TSC therapy was a feasible treatment for airway epithelial injury (Ghosh et al., 2017). Mouse stem cells were tested in adult C57Bl/6 mice while human stem cells were tested in immuno-compromised NOD/SCID/IL2Rγ KO (NSG) mice. We reported that transplanted TSC (both mouse and human) repopulated the tracheal epithelium when: 1) the airway sustained a *severe* airway epithelial injury which persisted 3 days; and 2) animals were treated prior to resumption of mucociliary clearance. TSC treatment of NA injured mice was effective when the treatment was instilled into the airways but not when delivered intravenously. The minimum effective dose was 2 x 10^6^ cells/kg. Histological analysis demonstrated that the transplanted TSC produced secretory (mouse Club cells; human mucus cells) and ciliated cells within 14 days and that the TSC-derived epithelium persisted for at least 40 days. This study established treatment parameters (host conditioning, timing, route, and dose) that are a prerequisite to development of UDSC therapy. Collectively, these data support the premise: TSC can be used to treat a chemical injury such as SM.

In this study, Cas9/RNP technology was used to generate human and rat B2M-KO-TSC. The ability of human B2M-KO-TSC to self-renew and generate differentiated epithelial cells was tested *in vitro*. Rat B2M-KO-TSC function was tested after transplantation into NA injured NSG mice. To evaluate B2M-KO-TSC as a possible MCM for SM induced lung disease, Fisher rat B2M-KO-TSC were used to treat SM challenged allogeneic Sprague Dawley rats. Efficacy and adverse treatment outcomes were evaluated over a 28-day time course.

## Materials and Methods

*Approvals: Human and animal study approvals:* The Institutional Review Board at Nationwide Children’s Hospital approved the human studies. Written informed consent and assent was obtained from every participant. All procedures involving animal use were approved by the Institutional Animal Care and Use Committee at Nationwide Children’s Hospital or Battelle Memorial Institute. Mice and rats were maintained in AAALAC-approved facilities and screened quarterly for pathogens.

*Isolation of human and rat TSC:* Human bronchial epithelial cells were recovered from explanted bronchial tissue using previously published methods (Hayes et al., 2018). Rat tracheal epithelial cells were recovered from Fisher rats by digestion with 0.15% Pronase (Sigma Cat#: PRON-RO, St Louis, MO; Walthan, MA) and processed as previously reported for mouse tracheal epithelial cells (You et al., 2002). Human and rat TSC were selected using the modified Conditional Reprograming Culture method (mCRC, (Reynolds et al., 2016).

*Generation of B2M-KO-TSC:* To determine the optimal electroporation conditions for TSC, we electroporated 5 x 10^5^ cells with 1 µg pmaxGFPTM Vector (Provided in P3 Primary Cell 4D-NucleofectorTM CAT# V4XP-3032) using Celetrix Biotechnologies (CB) Electroporator (CTX-1500A). Four electroporation conditions consisting of 400, 420, 440, and 450 volts (V) for 30 milliseconds (ms) were tested and it was determined that the 450V condition yielded the greatest number of viable green fluorescent protein (GFP) positive cells 24 h post-electroporation (data not shown).

For gene editing, single guide RNAs (sgRNA) that were complementary to the human or rat B2M gene were designed using a web-based design tool (Benchling.com) and tested separately. Cas9 reagents were introduced to passage 2 or 3 TSC as Cas9/RNP. The methodology for making Cas9/RNP complexes has been previously described (Naeimi Kararoudi et al., 2020). Briefly, a crRNA/trRNA complex was generated by combining 2.2 µl of Cas9 (crRNA, Alt-R^®^ crRNA, Integrated DNA Technologies (IDT), Inc., Coralville, Iowa), 2.2 µl of tracer RNA (trRNA, Alt-R^®^ Cas9 trRNA, IDT), and 5.6 µl IDT buffer in final volume of 10 µl. Next, 2 µl of sgRNA was incubated with 2 µl of pre-translated HiFi Cas9 (Alt-R^®^ S.p. HiFi Cas9 Nuclease V3, IDT Cat# 1081061) for 15 min at room temperature to form the Cas9/RNP complex. Next, 5x10^5^ cells per condition were washed twice with 1x phosphate buffered saline (PBS) and resuspended 20 µl of Celetrix electroporation buffer (combination of 10 µl A and 10 µl of B buffers, CB Cat# 1207). Cas9/RNP complex and 1 µl of 100 uM Electroporation Enhancer (IDT Cat# 1075916) were added to the cell suspension and transferred into electroporation cuvettes (CB Cat# 1207) and electroporated at 450V for 30 ms. The cells were using the mCRC method. One day after electroporation, irradiated NIH3T3 fibroblasts were added to the cultures at a density of 3.24 x 10^4^ cells/cm^2^. On culture day 5, TSC were recovered using the double-trypsinization method.

*Flow cytometry:* Human B2M wild type- (WT) and B2M-KO-TSC were resuspended at 10^7^ cells/ml in BSA staining buffer (BD Pharmingen Cat# 554657) and stained with PE-anti-B2M (Novus Biologicals Cat# NBPL-44523) and APC-anti-MHC-I (BD Pharmingen Cat# 555555) for 30 min on ice. Rat cells were resuspended as indicated above and stained with Novus-anti-B2M. Cells were washed twice, fixed with 10% neutral buffered formalin (NBF), and analyzed on a BD Pharmingen Fortessa-5 laser flow cytometer. Unstained cells served as the negative control and beads were used to set compensation. Following transplantation, rat B2M-KO-TSC were recovered from trachea or lung as previously reported (Ghosh et al., 2011b). The cytometer was calibrated using GFP beads (ThermoFisher Cat#A10514). Flow data were analyzed using FlowJo.

*Detection of off-targets gene editing events:* To identify the off-target effects of Cas9/RNP editing of the *B2M* gene in human TSC, published methods were used to conduct whole genome sequencing (Naeimi Kararoudi et al., 2020) and the data were analyzed using a novel bioinformatic pipeline called “Churchill” (Kelly et al., 2015). Briefly, isogenic wild type (WT)- and B2M-KO-TSC were sorted and genomic DNA (gDNA) was isolated using a Qaigen DNease Blood and Tissue kit (Cat# 69506). Whole genome sequencing was performed using Illumina HiSeq4000 platform at 2 x 150 bp read lengths to a depth of ∼30X coverage. The reads were aligned to the GRCh37 reference genome. The hits were filtered according to the following criteria: Coding sequence (missense or nonsense) or splice site variant, passed all filters (PASS), or clustered events. This approach reported exclusive single nucleotide polymorphisms and insertion-deletion mutations (indels).

*Stem cell frequency analysis: Human and rat* TSC were enumerated using the limiting dilution assay (Ghosh et al., 2013). The clone forming cell frequency times 1000 (CFCFx1000) was reported.

*Human TSC differentiation analysis:* TSC were plated onto collagen-coated 0.33 cm^2^ transwell membranes at 2 × 10^4^ cells per membrane as previously described (Reynolds et al., 2016). At confluence, the medium was changed to Half & Half differentiation medium (Malleske et al., 2018). On differentiation day 21, the cultures were fixed and stained. Nuclei were detected using 4′ 6-diamidino-2-phenylindole (DAPI), mucus cells were detected with rabbit-anti-MUC5B (1/100, (Seibold et al., 2013)), and ciliated cells were detected with mouse-anti-acetylated tubulin (1/8000, ACT, (Seibold et al., 2013)). Differentiation was quantified using a serological method (Reynolds et al., 2016) that meets the American Thoracic Society standard for assessment of histological data sets (Hsia et al., 2010).

*Gene expression analysis:* Human TSC were expanded or differentiated as indicated above. RNA was purified as previously reported (Ghosh et al., 2021). Time points are indicated in Results. RNA sequencing (RNAseq) and bioinformatics analysis were done as previously reported (Ghosh et al., 2021).

*Rat B2M-KO-TSC transduction*: Passage 4 rat B2M-KO-TSC were transduced with HIV-mPGK-EGFP-VSVG (Iowa Viral Vector Core, Cat# VVC-IOWA-7545) as previously reported (Jr et al., 2021). Briefly, two lots of 1.5 x 10^7^ cells were transduced with at a multiplicity of infection of 50 viral genomes/TSC (MOI 50:1). Transduced cells were cultured using the mCRC method. At passages 6–10, cells were recovered by the double trypsinization method. Enhanced green fluorescent protein (EGFP) expression was assayed by flow cytometry.

*NA transplantation assay*: NA was prepared as previously reported (Cole et al., 2010). A group of 8 female NOD/SCID/IL2R gamma chain-KO mice (NSG, JAX 005557) were treated with 300 mg/kg NA by intraperitoneal injection (Cole et al., 2010). On recovery days 2–4 the mice were treated with 1 ml sterile saline by subcutaneous injection. On post-NA treatment day 2, mice were anesthetized with isoflurane. Vehicle (0.03 ml PBS) or passage 6 EGFP-positive rat B2M-KO-TSC (10^6^ cells in 0.03 ml PBS) were delivered by intratracheal instillation. Body weight was determined daily for 6 days and every 2–3 days thereafter.

*Mouse lung histology*: On post-NA days 7 and 30, tracheal and lung tissue was recovered, fixed with NBF, and embedded in paraffin. The EGFP marker was detected by staining with chicken anti-EGFP (1/500, Aveslabs, Cat# GFP-1020) primary antibody and an Alexa-488 donkey anti-chicken secondary antibody (1/500, Jackson Immunoresearch, Cat# 703-545-155). The club cell marker SCGB1A1 was detected using a goat-anti-rat SCGB1A1 (1/500 for trachea, 1/10,000 for lung, (Stripp et al., 2002)). The ciliated cell marker acetylated tubulin (ACT) was detected using a mouse anti-ACT antibody primary antibody (1/8000, Sigma, Cat# T7451). The basal cell marker Keratin 5 was detected using a rabbit-anti-Keratin 5 primary antibody (1/1000, Invitrogen Cat#PA5-32465). The SCGB1A1, ACT, and Keratin 5 antibodies were detected using Alexa-594 labeled donkey anti-goat, donkey anti-mouse, or donkey anti-rabbit secondary antibodies (1/500, Jackson Immunoresearch, Cat# 705-585-147, Cat# 715-585-151, CAT#711-585-152). DNA was detected using DAPI. Tiled images of the entire tracheal and lung sections were acquired using a Leica Scanscope and Axiovision software.

*SM Exposure System*: SM is a federally regulated agent and SM exposure studies are conducted by a small number of certified laboratories. Battelle Memorial Institute is one such facility and utilizes a custom designed SM inhalation exposure system to simultaneously challenge up to 10 rats simultaneously to controlled SM vapor concentrations (Perry et al., 2021). SM vapor is generated by metering house air through a custom permeation vapor generator containing <10 ml of neat SM while a temperature-controlled aluminum block maintains the vapor generator at 50 C. The dilution airline is heat-traced to prevent vapor condensation. Target vapor concentrations are achieved by controlling air flow rate through the vapor generator, while block temperature and the dilution air flow rate (∼8.4 L/min) is kept constant. The generated SM exposure concentrations are monitored in near-real-time using a gas chromatograph/flame ionization detector and an inline gas sampling loop. Total inhaled dose (mg/kg) was calculated from the mean SM concentration, the total inhaled volume during exposure period, and the animal’s body weight from study day minus 1. In addition, animal exposure level (mg x min/m^3^) was calculated from the mean SM concentration multiplied by the exposure duration. In preparation for the present study, SM lethality was evaluated for both inhaled SM dose and exposure level (Perry et al., 2021) and showed that inhaled SM dose reduced variation in lethality values. This consistency allows for a more reproducible animal model of SM toxicity which is optimal for evaluating MCM.

*SM challenge:* Sixty male Sprague Dawley rats were used to evaluate Fisher rat B2M-KO-TSC as a treatment for SM-induced lung damage. Prior to challenge, animals were anesthetized with a ketamine/xylazine 80/10 gm/kg mixture administered intraperitoneally. Anesthesia was maintained by providing booster anesthetic doses of ketamine (16 mg/kg) when needed. After induction of sufficient anesthetic plane, endotracheal tubes (ET) were placed, and animals were put on a warming board in the sternal position until transfer to the inhalation system. The ET tube was a 16G 5 cm Termuno Surflo IV catheter that was trimmed to 4 cm. This catheter length was chosen so that the trachea would be exposed to SM. The tip of the catheter was positioned in the middle of the trachea cranial to the carina. Intubated animals were placed within the SM inhalation system and exposed to SM in the sternal position. During SM exposure, each animal’s respiration parameters were monitored in real-time via an in-line pneumotachometer, differential pressure sensors, amplifiers, and EMKA pulmonary data analysis software. Each animal had a targeted inhaled volume based on the target inhaled dose (0.8 mg/kg), SM concentration and animal body weight. The target SM concentration was 150 mg/m^3^ for all animal exposures. Upon reaching the target inhaled volume, the animals were disconnected from the inhalation system, ET tubes removed, and animals were placed in cages within the chemical fume hood where they remained until completion of the off-gassing period. A safe duration was defined as the period necessary to achieve off-gassing levels which were below the worker protection limit, 0.0004 mg/m^3^. After completion of off-gassing, animals were returned to their home cages. Animals were observed at least hourly during the off-gassing period and at least twice daily thereafter. Euthanasia occurred on 7-, 14-, and 28-day post-SM exposure.

*Rat B2M-KO-TSC treatment of SM challenged rats*: On post SM-challenge day 2, passage 6 EGFP-positive rat *B2M-KO-TSC* were recovered and resuspended in PBS at 1.6 x 10^7^ cells/ml. Viability was >99%. Treatment vehicle and B2M-KO-TSC were placed on wet ice and delivered to the exposure site at Battelle Memorial Institute. Animals were treated 48 ± 2 h post-SM challenge. Unanesthetized animals were placed in a restraining tube with the tail exposed and 4 x 10^7^ B2M-KO-TSC/kg or an equal volume of vehicle were delivered via intravenous tail injection. Treatment volumes for vehicle and rat B2M-KO-TSC ranged from 0.6 to 0.73 ml. Treatment administration over 15 s caused immediate lethality in 4 rats. The cause of death was not determined. Subsequently, treatment time was increased to 60 s and no adverse events were noted. Following treatment, the animals were returned to their cages.

*Longitudinal assessment of SM challenged rats*: Clinical observations were conducted daily and included assessment of live/dead, ataxia, rales, wheezing, respiratory distress, breathing rate (decreased, increased, rapid, forced abdominal, labored), lethargy, posture (hunched, prostrate), grooming (rough coat), fecal production, and vocalization. Body weights were collected at least once prior to challenge for use in determining randomization by group and on study day 0 for targeted inhaled volume determination. Post-challenge body weighs were recorded on study day 1, 3, 5, 7, 10, 14, 21, and 28. Oxygen saturation and heart rate data were collected using the MouseOx Plus small animal oximeter equipped with a rat collar sensor (Starr Life Sciences Corp). Measurements were performed in awake rats prior to challenge on study day 0 and then on study days 1, 3, 5, 7, 10, 14, 21, and 28. If non-error data was not obtained after 5-time-outs of the system then the collection was stopped and documented that data collection in the animal was unsuccessful.

*Post-mortem assessment of SM challenged rats*: Necropsies, bronchoalveolar lavage (BAL) collection, and tissue collection were performed on scheduled deaths. Surviving animals were euthanized by an injection of euthanasia solution (Euthasol) on post-challenge days 7, 14, and 28. Trachea and wet lung weights were collected first. Next, the right lungs were lavaged two consecutive times each with 5.0 ml cold phosphate buffered saline. The BAL fluid from the first and second lavages were pooled into one tube. The total volume, color and/or appearance was recorded. BAL samples were kept on wet ice until processing. Cells were collected by centrifugation at 2500 rpm for 5 min. The pellet was resuspended in PBS and the cells counted. Cytospin preparations were generated, stained with DiffQuick, and macrophages, neutrophils, lymphocytes, eosinophils, and basophils were enumerated. A second cytospin preparation was stained for EGFP, Keratin 5, SCGB1A1, and ACT using the methods presented above. BAL protein determination using the PierceT BCA Protein Assay kit (Cat# 23227, Pierce). Protein targets were assayed by ELISA. Kits from R&D Systems were used to quantify Total MMP-9 (Cat#: RMP900); TGFβ1 (Cat#: DB100B); PDGF-AB (Cat#: DY8460-05). A kit from Molecular Innovations was used to quantify total PAI-1 (Cat#: RPAIKT-TOT-1KIT). The trachea and right lungs were explanted into PBS. Trachea and right lungs were digested with dispase/collagenase/trypsin and analyzed for EGFP-positive cells by flow cytometry using previously reported methods (Ghosh et al., 2011b). The left lung was inflation-fixed at 10 cm water pressure with NBF for 20 min, excised, and immersion fixed in NBF overnight at 4°C. Lung tissue was embedded in paraffin, sectioned at 5 µm, and stained with hematoxylin and eosin (H&E).

*Statistical methods*: Descriptive statistics for TSC analyses and *in vivo* measurements (body weights, terminal wet lung weights, BAL fluid total protein content, counts of cells and differentials from lung lavage fluid, BAL protein ELISAs, and pulse oximetry) were generated. For each endpoint, ANOVA models were fit to the data to compare the treatment group to the control group for each day. Additionally, change from baseline was summarized for body weights and pulse oximetry measurements. ANOVA models were fit to compare these changes across groups: specific post-hoc tests are noted in figure legends. Approximate t-tests were used to assess changes from baseline for each group and each timepoint. Survival rates were summarized by group and Kaplan-Meier curves were estimated and plotted for each group. The mortality rates between the treatment group and the control group were compared using Boschloo’s test on study days 7, 14, and 28. Log rank tests were performed to compare the survival distributions between the groups.

## Results

**FIGURE 1:**
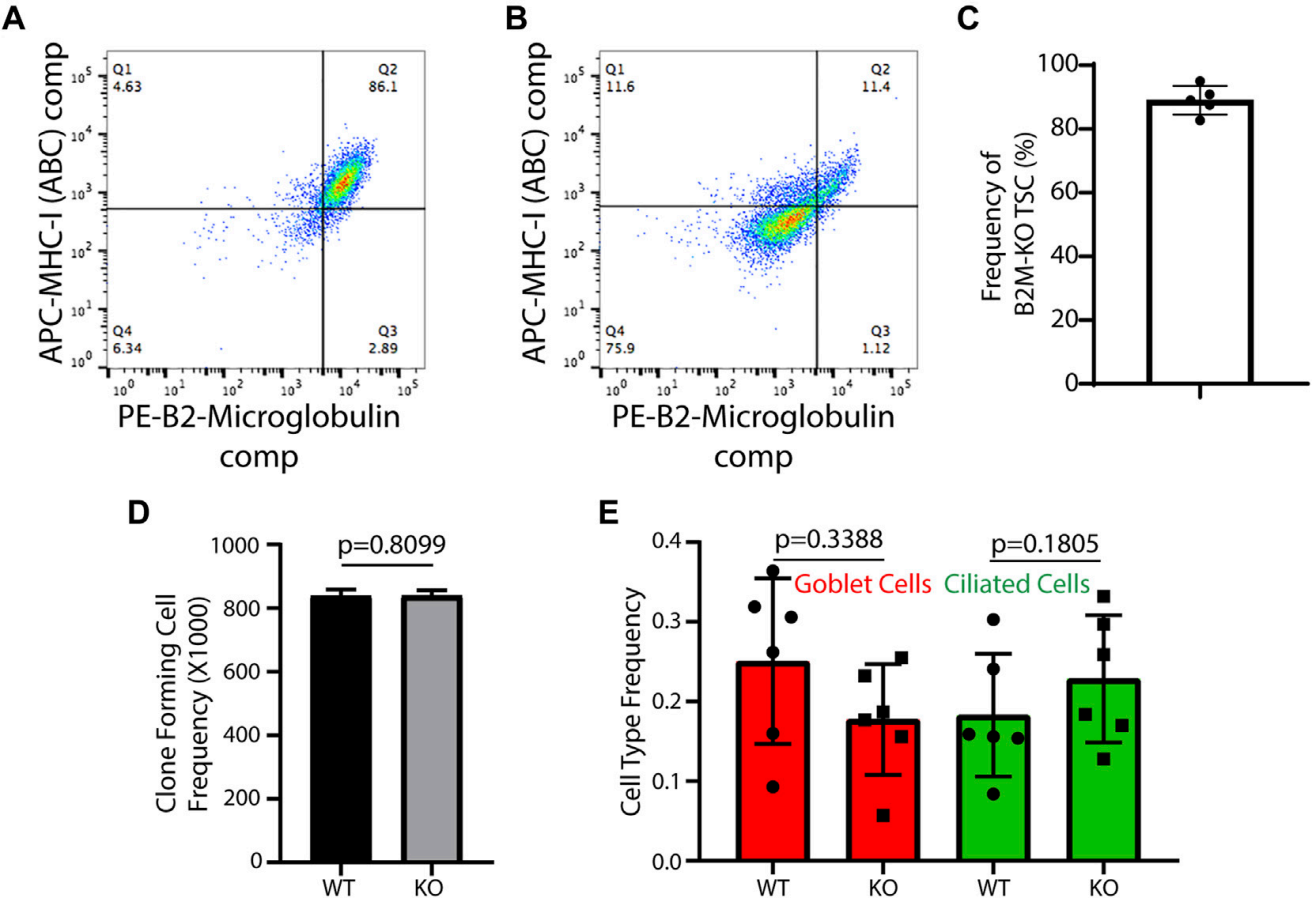
Development and functional testing of human B2M-KO-TSC. **(A,B)** Isogenic wild type TSC **(A)** and Cas9/RNP treated human TSC **(B)** were assayed for expression of B2M and MHC-1 by flow cytometry. **(C)** The frequency of B2M-KO-TSC was determined for 5 donors. The mean ± standard deviation (*n* = 5) is reported. **(D)** Self-renewal potential of wild type-TSC and B2M-KO-TSC was determined using the clone forming cell frequency assay. The mean ± standard deviation (*n* = 4) is reported. **(E)** The differentiation potential of wild type- and B2M-KO-TSC was compared using the air-liquid-interface culture model. Mucus cells were identified by staining for Muc5B and ciliated cells were identified by staining for acetylated tubulin. The mean ± standard deviation (*n* = 6) is reported.

*Generation of human B2M-KO-TSC:* Following Cas9/RNP treatment, TSC were expanded using the mCRC method. At passage 3 or 4, expression of B2M and MHC-I in untreated cells (Figure 1A) and Cas9/RNP treated cells (Figure 1B) were analyzed by flow cytometry. An analysis of 5 donors demonstrated that 89 ± 4% of edited cells were B2M-neg/MHC-I-neg (Figure 1C).

*Human B2M-KO-TSC function:* The frequency of clone forming cells was assayed using the CFCF assay. An analysis of isogenic WT- and B2M-KO-TSC from 4 donors showed that TSC frequency was similar in the two samples (Figure 1D). Consistent with this finding, the growth kinetics, clone area, and burst size did not vary between WT- and B2M-KO-TSC. The ability of WT- and B2M-KO-TSC to differentiate was assayed using the air-liquid-interface culture method. This study demonstrated that the frequency of mucus and ciliated cells did not vary by genotype (Figure 1E). Collectively, these data indicated that the editing process did not alter TSC self-renewal or multilineage differentiation.

*Analysis of off-target edits:* Whole genome sequencing and Churchill analysis of isogenic human WT- and B2M-KO-TSC identified 331 variants. However, only 16 genes, including B2M, were highly affected by Cas9/RNP treatment and were exclusive to the B2M-KO-TSC (Table 1). Two mutations were classified as multiallelic and were in the target gene, B2M. Three mutations were categorized as Pass All Filters (PASS) (Kelly et al., 2015) which indicates that the mutation could be identified as an event that was exclusive to the Cas9 edited cells. This category included mutations in B2M, KMT2A, and MT-ND2. COSMID analysis did not predict KMT-2 or MT-ND2 as off-target sites, potentially due to the modest similarity between the gRNA and gene sequences (Supplementary Figure S1A). These data illustrate the ability of Churchill to identify off-target mutations that may not be predicted by *in situ* approaches. Overall, these data demonstrate the low off-target effect of Cas9/RNP gene editing in TSC.

**TABLE 1:** Whole genome sequencing and Churchill analysis of off target edits in B2M-KO TSC.

Gene	Chr^1^	Start	Allele frequency in WT cells (%)	Allele	Reference	Alternate	Filter	Loc In gene	Effect	Impact
				Frequency	Sequence	Sequence				
				In B2M KO (%)						
B2M	15	45003766	0.00	40.00	GC	G	Multiallelic	Coding sequence	Frame shift	HIGH
B2M	15	45003766	0.00	50.00	GCT	G	Multiallelic	Coding sequence	Frame shift	HIGH
B2M	15	45003751	0.00	20.00	CGC​TCC​GTG​GCC​TTA​GCT​GT	C	PASS	Coding sequence	Frame shift	HIGH
KMT2	11	118374591	0.00	6.98	A	T	PASS	Coding Sequence	Stop Gain	HIGH
MT-ND	MT^2^	5086	0.00	0.6	CTA	C	PASS	Coding Sequence	Frame Shift	HIGH
DUSP23	1	159750975	0.00	18.75	C	T	Clustered events	Coding sequence	Stop gain	HIGH
PCDHA8	5	140222148	0.00	13.64	CCG	C	Clustered events	Coding sequence	Frame shift	HIGH
PCDHA8	5	140222157	0.00	9.09	A	ACC	Clustered events	Coding sequence	Frame shift	HIGH
PCDHA8	5	140222156	0.00	8.70	G	GACCAC	Clustered events	Coding sequence	Frame shift	HIGH
ARID1A	1	27106993	0.00	12.50	A	AG	Clustered events	Coding sequence	Frame shift	HIGH
ARID1A	1	27106418	0.00	7.14	TCC​TGG​GCA​AGC​TGA​TCC​TG	T	Clustered events	Coding sequence	Frame shift	HIGH
RBM6	3	50099517	0.00	12.12	G	GA	Clustered events	Coding sequence	Frame shift	HIGH
RBM6	3	50099537	0.00	6.67	C	CAA​AGT​TTC​AAG​AAA​GTA​CCA​GT	Clustered events	Coding sequence	Frame shift	HIGH
RBM6	3	50099543	0.00	6.25	T	G	Clustered events	Splice site		HIGH
RBM6	3	50004957	7.84	9.30	T	TC	Clustered events	Coding sequence	Frame shift	HIGH
SLC22A2	6	160679523	0.00	10.34	A	T	Clustered events	Coding sequence	Stop gain	HIGH
SLC22A2	6	160679523	0.00	9.68	A	T	Clustered events	Coding sequence	Stop gain	HIGH
SLC22A2	6	160679525	0.00	9.68	AC	A	Clustered events	Coding sequence	Frame shift	HIGH
SLC22A2	6	160679534	2.78	6.31	G	GCTGAGGAA	Clustered events	Coding sequence	Frame shift	HIGH
ILK	11	6629398	0.00	9.09	TG	T	Clustered events	Coding sequence	Frame shift	HIGH
ILK	11	6629441	0.00	7.41	G	GCT​GTT​GCA​ATA​CAA​GAC​T	Clustered events	Splice region	Non- frame shift insertion	HIGH
								Coding sequence		
RGS5	1	163122406	0.00	7.50	A	ATG	Clustered events	Coding sequence	Frame shift	HIGH
RGS5	1	163122403	0.00	7.14	GGC	G	Clustered events	Coding sequence	Frame shift	HIGH
ZBTB6	9	125674108	0.00	7.32	T	A	Clustered events	Coding sequence	Stop gain	HIGH
SCYL2	12	100711705	0.00	7.32	T	C	Clustered events	Splice site		HIGH
KMT2A	11	118373872	2.27	8.82	GA	G	Clustered events	Coding sequence	Frame shift	HIGH
KMT2A	11	118373868	2.33	8.11	AT	A	Clustered events	Coding sequence	Frame shift	HIGH
KMT2A	11	118373855	2.27	7.50	T	TCTAC	Clustered events	Coding sequence	Frame shift	HIGH
KMT2A	11	118373861	2.38	7.14	CGA	C	Clustered events	Coding sequence	Frame shift	HIGH
KMT2A	11	118344414	4.44	6.25	GA	G	Clustered events	Coding sequence	Frame shift	HIGH
KMT2A	11	118344411	4.55	6.12	A	AG	Clustered events	Coding sequence	Frame shift	HIGH
GBE1	3	81698016	0.00	6.67	T	A	Clustered events	Coding sequence	Stop gain	HIGH
DNAJC27	2	25174366	0.00	6.00	TGG	T	Clustered events	Coding sequence	Frame shift	HIGH
DNAJC27	2	25174370	0.00	4.08	G	GCC	Clustered events	Coding sequence	Frame shift	HIGH
HNRNPF	10	43883157	0.00	5.97	AC	A	Clustered events	Coding sequence	Frame shift	HIGH
HNRNPF	10	43883192	3.23	7.84	G	T	Clustered events	Coding sequence	Stop gain	HIGH
HNRNPF	10	43883250	2.94	4.26	TGC	T	Clustered events	Coding sequence	Frame shift	HIGH
HNRNPF	10	43882242	4.26	5.56	GTTGAA	G	Clustered events	Coding sequence	Frame shift	HIGH
HNRNPF	10	43882276	5.88	5.56	G	A	Clustered events	Coding sequence	Stop gain	HIGH
HNRNPF	10	43882217	4.88	4.44	A	C	Clustered events	Coding sequence	Stop gain	HIGH

1. Chr: Chromosome

2. MT, mitochondrial

Shading indicates targeted (B2M) and off-target mutations KMT2 and NT-ND2)

Clustered events are sequencing artifacts or low-quality variant calls

Mutations categorized as clustered events indicate a sequencing artifact or low-quality variant call. While clustered events are usually removed, we reported them as they were unique and exclusive to the Cas9-edited cells. Most of these putative mutations were in coding sequence except for one splice site edit. Nine of 16 putative mutations resulted in a frame shift and 5 of 16 mutations were categorized as stop/gain which introduces a stop codon or a change that is likely result in a gain of function resulting from removal of a stop codon. We also evaluated expression of the targeted genes during TSC proliferation and differentiation *in vitro* (Supplementary Figure S1). Six of 16 genes were highly transcribed in proliferating TSC and only 2 of 16 genes increased expression as a function of time in differentiating TSC.

**FIGURE 2:**
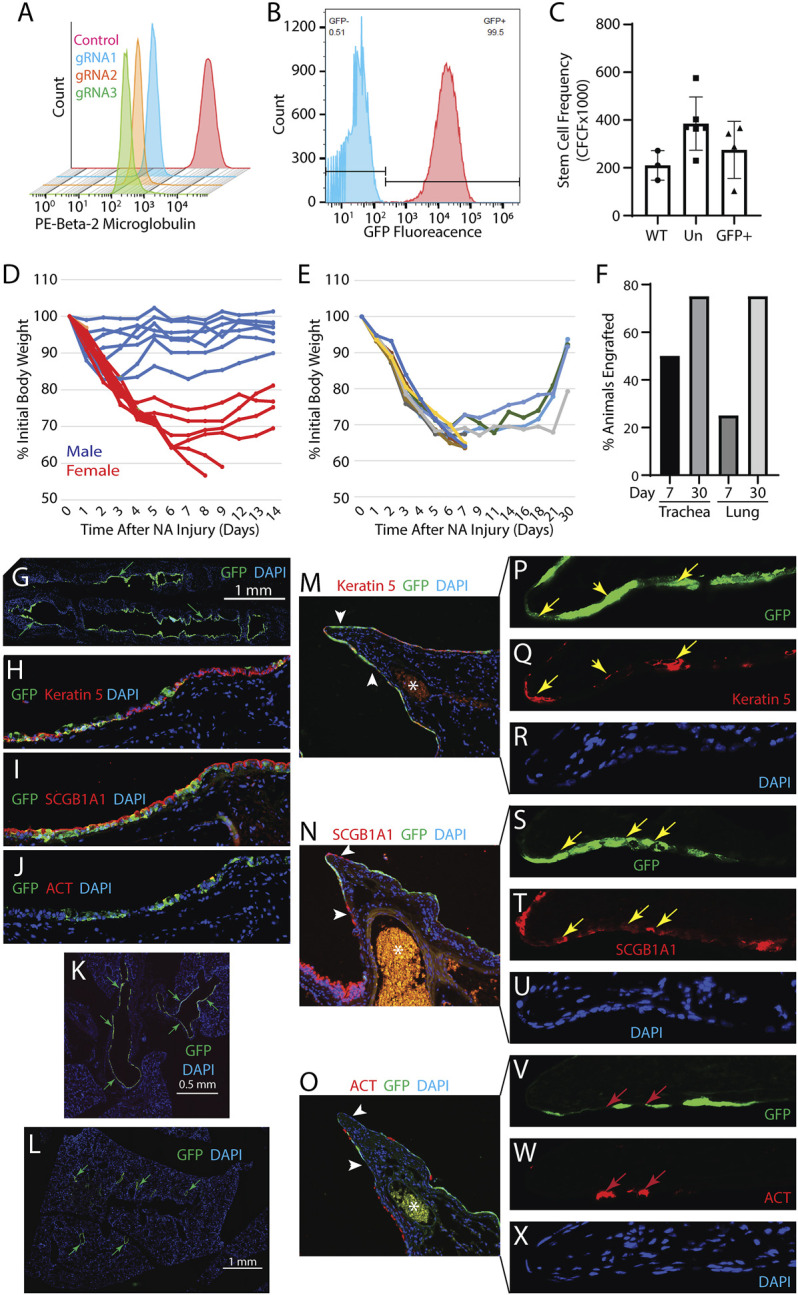
Development and functional testing of rat B2M-KO-TSC. **(A)** Isogenic wild type TSC (control, red histogram) and Cas9/RNP treated Fisher rat TSC (gRNA1-3, blue, yellow, and green histograms) were assayed for expression of B2M by flow cytometry. **(B)** B2M-KO-TSC were transduced with an EGFP-expressing lentivirus and assayed by flow cytometry. Unstained control, blue histogram and native EGFP fluorescence, red histogram. **(C)** The frequency of TSC in wild type (WT) control, untransduced B2M-KO-TSC (Un), and transduced B2M-KO-TSC (GFP+) was compared using the clone forming cell frequency assay. The mean ± standard deviation (*n* = 3–6) is reported. **(D)** Evaluation of sexual dimorphism in the NA injury model. Male (blue lines) and female (red lines) mice were treated with 300 mg/kg NA and body weight was determined over 14 days. Changes in body weight are reported as the percent of initial body weight. Each line represents an individual mouse. **(E)** Body weight change in female NSG mice that were challenged with 300 mg/kg NA and treated with rat B2M-KO-TSC. Changes in body weight are reported as the percent of initial body weight. Each line represents an individual mouse. **(F)** Analysis of tracheal and lung engraftment in 4 mice on day 7 and 4 mice on day 30. Tracheal and lung tissue sections were immunostained for EGFP and scored for the presence/absence of EGFP+ cells. 12 sections spanning ∼60 μm of tissue were evaluated. The bars indicate the percentage of mice that were engrafted. **(G–J)** Engraftment of the tracheal epithelium by rat B2M-KO-TSC. Distribution of EGFP+ cells (green) in the trachea **(G)**. Nuclei are stained with DAPI. Representative of 3 mice on day 30. Immunostaining for EGFP (green, **(H–J)**) and Keratin 5 (red, **(H)**), or SCGB1A1 (red, **(I)**), or acetylated tubulin (ACT, red, **(J)**). Nuclei are stained with DAPI (blue). Magnification equals 200X. K-X. Engraftment of the intrapulmonary airway epithelium by rat B2M-KO-TSC. Distribution of EGFP+ cells (green, arrows) in the lungs showing the mainstem bronchi **(K)** and intrapulmonary airways **(L)**. Representative of 3 mice on day 30. The third branchpoint was evaluated for EGFP+ rat B2M-KO-TSC (green, **(M–O)**). 16 sections spanning ∼80 μm of tissue were evaluated. Immunostaining for EGFP and Keratin 5 (red, **(M)**), or SCGB1A1 (red, **(N)**), or acetylated tubulin (ACT, red, **(O)**). Nuclei are stained with DAPI (blue). Asterisks indicates a blood vessel containing autofluorescent erythrocytes. Arrows indicate that region presented in panels **(P–X)**). Magnification in M-O equals 200x. Magnification in P-X equals 800x. Yellow arrows indicate EGFP+ cells that co-express Keratin 5 **(P, Q)** or SCGB1A1 **(S, T)**. Red arrows indicate EGFP- cells which are positive for ACT.

*Generation and function of EGFP labeled rat B2M-KO-TSC:* Rat TSC were edited at the B2M locus using a Cas9/RNP, 3 different gRNA, (gRNA1: 5′ TCA​GCA​AGG​ACT​GGT​CTT​TC, gRNA2: 5′ GAC​AAG​CAC​CAG​AAA​GAT​CA and gRNA3: 5′ CAC​CGA​GCG​AGC​CAT​CGT​GC) and the optimized electroporation protocol. Flow cytometry analysis demonstrated that 90–95% of the cells were B2M negative (Figure 2A). Since all three gRNA were equally effective, subsequent experiments used gRNA1. MHC-I expression was not assayed due to a lack of rat-specific reagents. Passage 4 rat B2M-KO-TSC were transduced with a EGFP expressing lentiviral vector and flow cytometry demonstrated that 99.4% of cells were EGFP-positive (Figure 2B). A serial passage study demonstrated that B2M-KO-TSC maintained high expression of EGFP over 5 consecutive passages (∼10 population doublings, data not shown). The self-renewal potential of EGFP-tagged rat B2M-KO-TSC was tested using the CFCF assay. The CFCF for WT TSC was similar to that of un-transduced, and EGFP-positive B2M-KO-TSC (Figure 2C).

*Optimization of NA transplantation model:* We previously reported that human or mouse TSC repopulated the tracheal epithelium of NSG mice (Ghosh et al., 2017). To determine if the transplantation studies could use both sexes, groups of 8 male and 8 female NSG mice were treated with 300 mg/kg NA and body weight was followed for 14 days (Figure 2D). This study demonstrated that female NSG mice were more sensitive to NA than male mice and justified single sex studies using female mice.

*Engraftment of rat* B2M-KO-TSC*:* The ability of EGFP-tagged rat B2M-KO-TSC to repopulate the lung was evaluated using the NA transplantation model. On NA recovery day 2, 33 x 10^6^ EGFP-positive rat B2M-KO-TSC/kg (∼10^6^ cells/mouse) were delivered by intratracheal instillation. Body weight change in B2M-KO-TSC treated mice (Figure 2E) was similar to that observed in untreated mice (Figure 2D). On recovery days 7 and 30, EGFP tagged rat B2M-KO-TSC and their descendants were detected by immunostaining for EGFP. On post-NA day 7, 50% of rat B2M-KO-TSC treated mice had EGFP+ cells in the tracheal epithelium and 25% had EGFP+ cells in the intrapulmonary airways. On post-NA day 30, 75% of mice had EGFP+ cells in the tracheal epithelium and 75% had EGFP+ cells in the intrapulmonary airways (Figure 2F).

Histological analysis detected EGFP+ cells throughout the tracheal epithelium (Figure 2G). Many of these patches contained cells which expressed the basal cell marker Keratin 5 (Figure 2H) or the club cell marker SCGB1A1 (Figure 2I). Host-derived regions of the epithelium contained few ACT+ ciliated cells and indicated an ongoing repair process. Consistent with this finding, occasional EGFP+ cells co-expressed the ciliated cell marker ACT (Figure 2J) while most engrafted cells were ACT-negative. These data indicated that rat B2M-KO-TSC retained their ability to undergo multilineage differentiation when engrafting the tracheal epithelium.

In contrast with our previous report (Ghosh et al., 2017), rat B2M-KO-TSC also engrafted the intrapulmonary airways (Figures 2K–O). EGFP+ cells were in large patches along the main axial pathway including the external lobar bronchus and bronchiolar epithelium (Figures 2K–L). EGFP+ cells were not detected in the terminal bronchiolar or alveolar epithelium. EGFP+ cells within the intrapulmonary epithelium co-expressed Keratin 5 (Figures 2M,P–R) or SCGB1A1 (Figures 2N,S–U). Like the tracheal epithelium, host-derived regions of the intrapulmonary airways contained few ACT+ ciliated cells and ACT+ cells were not identified in EGFP+ engrafted regions (Figures 2O,V–X). These data indicated that the rat B2M-KO-TSC maintained their ability to generate club cells when engrafting the bronchiolar epithelium.

*SM challenge and rat B2M-KO-TSC treatment:* The experimental design for SM challenge and B2M-KO-TSC treatment is shown in Table 2. To detect both beneficial and detrimental effects of the B2M-KO-TSC treatment, a target inhaled SM dose which resulted in 50% death on day 28 was selected. A previous study (Perry et al., 2021) of SM survival reported that the inhaled LD_50_ on day 28 was 0.8 mg/kg. The respiratory parameter results for SM challenged rats in the present study are shown in Table 3. The SM target inhaled dose, actual inhaled dose, mean concentrations achieved, calculated exposure durations for each group, and exposure level (mg x min/m^3^) are shown in Table 4. The mean SM concentrations for each group ranged from 148.9 to 153.1 mg/m^3^, with mean exposure durations ranging from 9.98 to 21.48 min. The mean inhaled dose for each group ranged from 0.80 to 0.82 mg/kg compared to group mean exposure levels ranging from 1708 to 2361 mg x min/m^3^. These data indicate that the actual inhaled SM doses for all animals were within 4% of the target dose of 0.8 mg/kg.

**TABLE 2:** Experimental design: SM challenge and vehicle or B2M-KO-TSC therapy.

Group	SM target dose	Therapy	Euthanasia day	Number of rats
1	0.8 mg/kg	Vehicle	7	10
2			14	10
3			28	10
4		B2M-KO-TSC^1^	7	10
5			14	10
6			28	10
Number of Replacement Rats				10
Total Number of Rats				70

1. 4 x 10^7^ B2M-KO-TSC/kg given 48 ± 2 h post SM challenge

**TABLE 3:** Animal respiratory parameter results.

Group	Animal IDs	Actual inhaled volume (L)	Tidal volume (ml)	Respiratory rate (bpm)	Minute volume (ml/min)
		Mean ± SD			
1	029, 040, 054, 055, 065, 068, 075, 089, 765, 2889	1.58 ± 0.08	1.50 ± 0.18	85 ± 9	128 ± 21
2	028, 030, 031, 034, 048, 051, 064, 083, 086, 2895	1.63 ± 0.09	1.56 ± 0.24	82 ± 13	126 ± 22
3	033, 038, 039, 045, 050, 056, 058, 070, 079, 2884	1.61 ± 0.06	1.45 ± 0.19	80 ± 6	115 ± 14
4	027, 041, 060, 069, 073, 081, 764, 2876, 2885, 2890	1.59 ± 0.06	1.51 ± 0.18	73 ± 9	109 ± 17
5	022, 049, 063, 066, 067, 071, 080, 2879, 2896, 2897	1.58 ± 0.07	1.48 ± 0.17	73 ± 10	106 ± 7
6	032, 036, 044, 082, 2877, 2878, 2881, 2883, 2886, 2891	1.54 ± 0.16	1.73 ± 0.13	80 ± 8	139 ± 18

**TABLE 4:** SM Inhaled Dose vs Concentration.

Group	Target inhaled dose (mg/kg)	Mean calculated inhaled dose (mg/kg)	Mean SM concentration (mg/m^3^)	Mean exposure duration ± SD (min)	Mean concentration x time ± SD (mg x min/m3)
1	0.8	0.82	153.1	12.9 ± 2.2	1978 ± 342
2	0.8	0.81	150.7	13.6 ± 3.0	2053 ± 458
3	0.8	0.82	152.0	14.5 ± 1.9	2199 ± 292
4	0.8	0.81	151.4	15.1 ± 2.3	2288 ± 354
5	0.8	0.82	152.2	15.5 ± 1.3	2361 ± 202
6	0.8	0.80	148.9	11.5 ± 1.0	1708 ± 147

*Lung histology in SM-challenged rats:* Histological analysis of lung sections from rats treated with vehicle or B2M-KO-TSC showed similar airway pathology (Supplementary Figure S2). On day 7, the bronchial epithelium was denuded in 5/5 vehicle-treated and 8/8 B2M-KO-TSC-treated rats. Bronchiolar epithelial pathology exhibited two subtypes. In each animal, approximately one-quarter of bronchiolar airways had an intact epithelium; whereas the remaining airways were either denuded or partially denuded. In damaged bronchiolar airways, epithelial sloughing or necrosis was noted. This pattern was observed in 5/5 vehicle-treated and 5/8 B2M-KO-TSC-treated rats. In the 3 remaining B2M-KO-TSC-treated rats, large regions of the bronchiolar epithelium were denuded but smaller bronchiolar airways were lined by a simple cuboidal epithelium. Terminal bronchiolar epithelial pathology also exhibited two categories. Approximately half of these regions had an intact epithelium, while the epithelium was necrotic in the remaining regions. This pattern was observed in 5/5 vehicle-treated and 6/8 B2M-KO-TSC-treated rats. The terminal bronchiolar epithelium in the 2 remaining B2M-KO-TSC-treated rats included regions which were normal or occluded with debris. These data indicate that SM-challenge caused severe airway epithelial damage and that little epithelial repair occurred through day 7.

*Analysis of tracheal and lung engraftment:* Flow cytometry was used to evaluate engraftment of rat B2M-KO-TSC on post-SM challenge days 7, 14, and 28 (Supplementary Figure S3, Supplementary Table S1). All events detected by the side scatter (SSC) and forward scatter (FSC, Supplementary Figure S3B) were included to ensure that rare events were not missed. Analysis of tracheal cells on day 7 (4.31 x 10^5^ to 5.01 x 10^5^ cells) detected ∼2–7% of cells with a green autofluorescence value which was equivalent to that of EGFP-labeled B2M-KO-TSC (Figure 2B, Supplementary Table S1). The frequency of EGFP+ cells in rats that were treated with B2M-KO-TSC ranged from ∼5–10% and was not different from that detected in vehicle-treated rats. Similar analyses on days 14 (6.33 x 10^4^–6.47 x 10^4^ cells) and day 28 (5.16 x 10^4^–2.73 x 10^5^ cells) also failed to detect EGFP+ cells in B2M-KO-TSC treated rats. To evaluate engraftment of the lung, 5 x 10^5^–2.5 x 10^6^ cells were evaluated by flow cytometry on post-challenge days 7 and 28. All events detected by the side scatter (SSC) and forward scatter (FSC, Supplementary Figure S4B) were included to ensure that rare events were not missed. These studies did not detect EGFP+ cells in rats that were treated with B2M-KO-TSC (Supplementary Figure S4, Supplementary Table S1). These data indicate that any engrafting rat B2M-KO-TSC were lost by the first assay time point, day 7.

**FIGURE 3:**
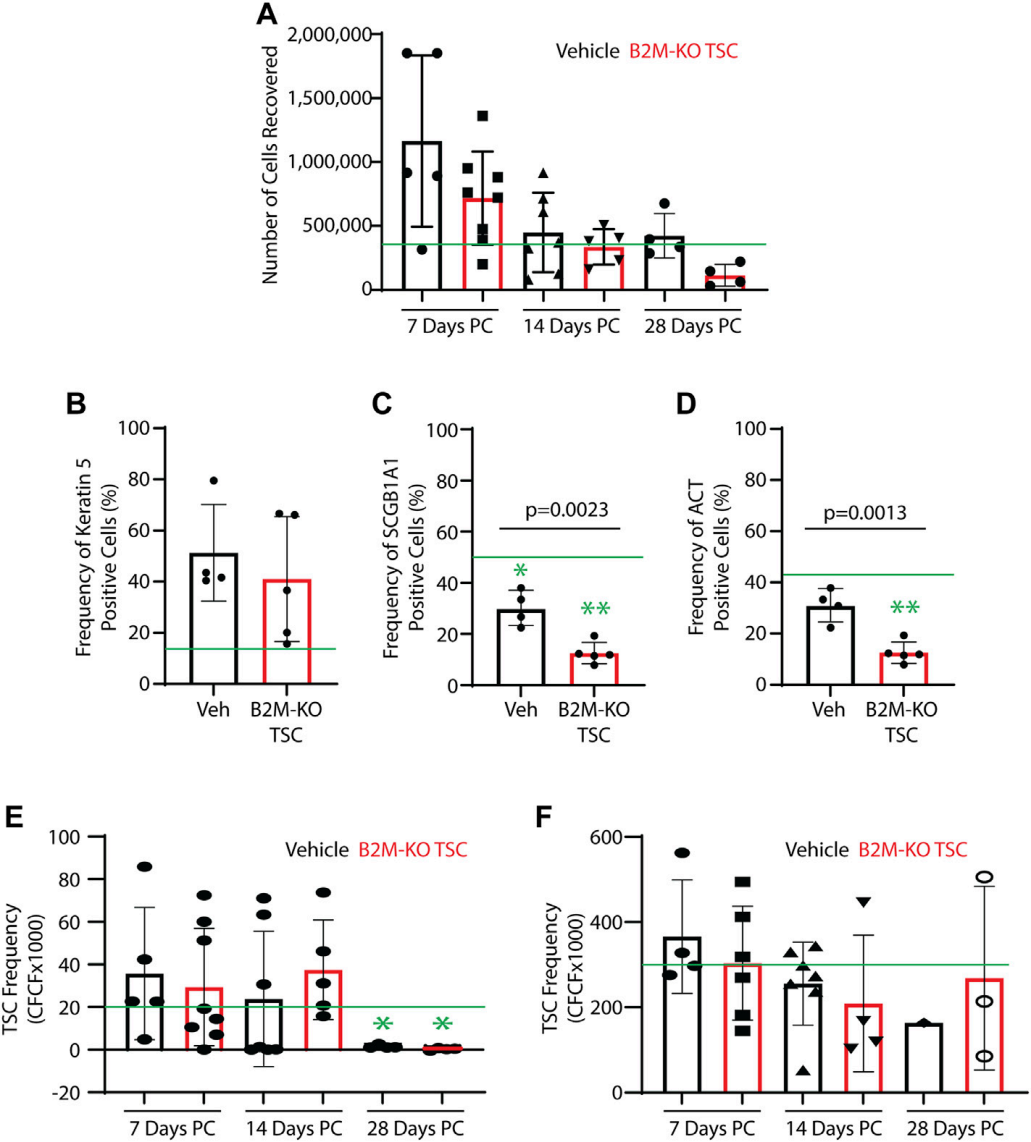
Impact of SM challenge on the tracheal epithelium. Trachea from SM-challenged rats that were treated with vehicle (Veh) or B2M-KO-TSC were digested to a single cell suspension. **(A)** Cell recovery from SM-challenged rats that were treated with vehicle or B2M-KO-TSC. Cells were recovered on post-SM-challenge days 7, 14, or 28. Mean ± standard deviation, *n* = 4–8. The green line indicates the average cell yield from normal rats (*n* = 6). **(B–D)** On PC day 7, cytospin preparations were immunostained for cell type specific markers. The frequency of Keratin 5+ basal cells **(A)**, SCGB1A1+ club cells **(B)**, and acetylated tubulin-positive (ACT+) cells **(C)** was determined. Mean ± standard deviation, *n* = 4–5. *p <0.05, **p <0.01 relative to untreated control. **(E, F)** Self-renewal potential of TSC recovered from vehicle and B2M-KO-TSC treated SM-challenged rats was determined using the clone forming cell frequency assay at passage 0 **(E)** and passage 1 **(F)**. The mean ± standard deviation (*n* = 4–8) is reported. The green line indicates TSC frequency in tracheal cells from unchallenged rats. *p <0.05.

*Impact of SM challenge and rat B2M-KO-TSC treatment on the TSC pool*: The number of cells recovered from protease-digested trachea exhibited a great deal of variability across time and treatment group. None of these values was different from normal Sprague Dawley rats (Figure 3A). To determine if SM challenge/vehicle treatment altered the frequency of tracheal basal, club, or ciliated cells on post-SM challenge day 7, cytospin preparations were stained for basal cells (Keratin 5), club cells (SCGB1A1), or ciliated cells (acetylated tubulin). Relative to normal control, SM challenge/vehicle treatment did not alter the frequency of basal cells (Figure 3B), decreased the frequency of club cells (Figure 3C), and did not alter the frequency of ciliated cells (Figure 3D). SM-challenge/B2M-KO-TSC treatment did not alter the frequency of basal cells relative to normal control or vehicle treatment (Figure 3B). However, SM-challenge/B2M-KO-TSC treatment decreased the frequency of club (Figure 3C) and ciliated cells (Figure 3D) relative to normal control and vehicle treatment.

TSC function in the trachea was examined using single cell isolates and the mCRC method. TSC colonies were identified in all passage 0 cultures and 25 of 33 samples could be passaged. TSC frequency was quantified using the clone forming cell frequency assay. TSC frequency in the vehicle and rat B2M-KO-TSC groups was similar to normal control on days 7 and 14 but was significantly decreased in both groups at on day 28 (Figure 3E). TSC frequency did not vary by treatment at any time point. At passage 1, TSC frequency was normal and did not vary with time or treatment (Figure 3F). Consistent with previous reports that rat basal cells are normally restricted to the trachea and bronchi, clone forming TSC were not detected in the lung tissue from vehicle or rat B2M-KO-TSC treated rats (data not shown). These data indicate that the tracheal TSC pool was depleted in SM-challenged rats on day 28 and that rat B2M-KO-TSC treatment did not reverse this process.

**FIGURE 4:**
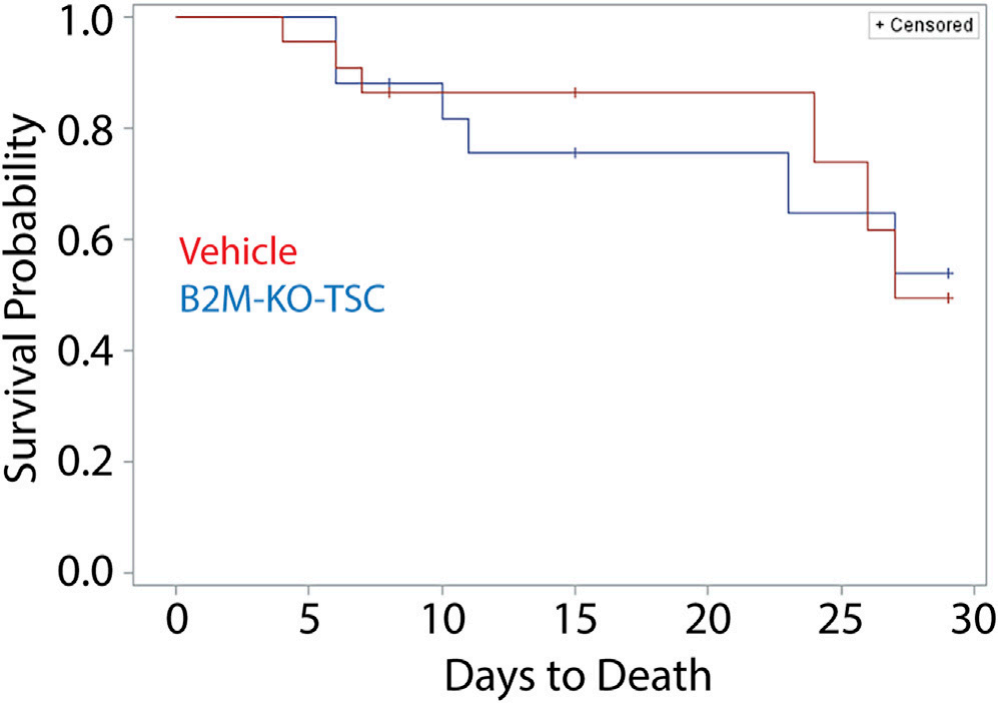
Mortality of SM-challenged rats that were treated with vehicle or rat B2M-KO-TSC. A Kaplan Meier plot comparing survival probability for vehicle-treated (red line), and rat B2M-KO-TSC-treated (blue line) rats. The plot was censored to factor in scheduled removal of animals at terminal timepoints of 7-day, 14-day, and 28-day post SM-challenge (tick marks).

*Impact of rat B2M-KO-TSC treatment on survival:* A Kaplan-Meier plot was used to compare survival in the vehicle- and rat B2M-KO-TSC-treated animals (Figure 4). Survival to day 28 was similar in the two groups. A two-sided Boschloo’s test was used to evaluate survival at each terminal time point. No significant differences were observed: day 7, *p* = 1.0000; day 14, *p* = 0.04128; and day 28 *p* = 1.0000. A log-rank analysis of mortality by treatment found a *p*-value of 0.8935 and was not significant. These data indicate that B2M-KO-TSC treatment did not alter survival over 28 days.

*Impact of rat B2M-KO-TSC treatment on SM induced lung damage*: The vehicle- and rat B2M-KO-TSC-treated groups did not differ in clinical observation onset, severity, or duration (data not shown). Respiratory abnormalities were the most common clinical finding. Changes including altered breathing (increased, decreased, or labored) were prevalent in both groups, were observed within 48 h of challenge, and persisted through death or scheduled termination for most of the animals. Other respiratory abnormalities included wheezing, rales, forced abdominal breathing, open mouth breathing and respiratory distress. These respiratory abnormalities did not vary by treatment or time.

**FIGURE 5:**
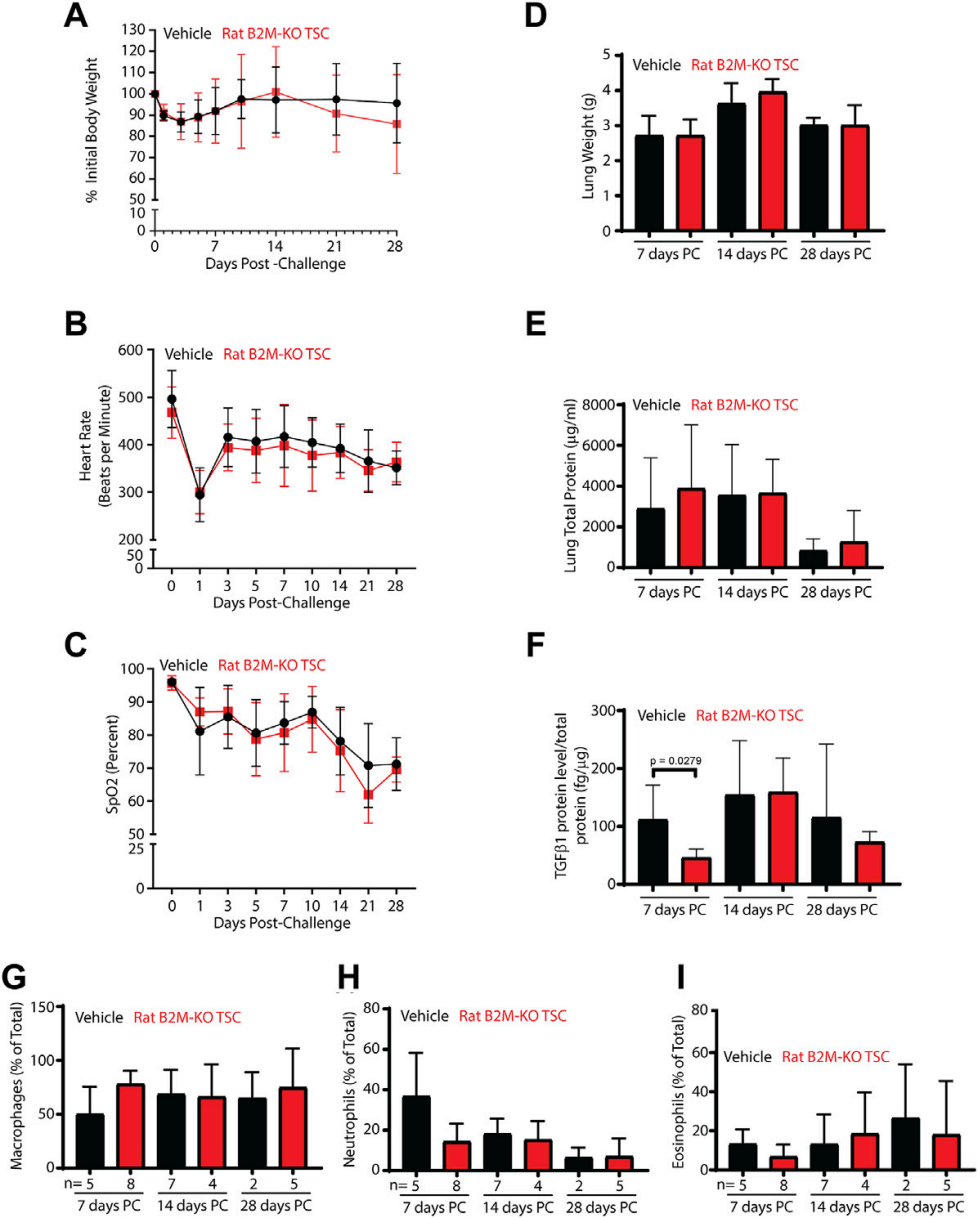
Impact of B2M-KO-TSC treatment on SM induced lung damage. **(A)** Body weight before SM-challenge and on post-SM-challenge days 1–28 is reported as a percentage of initial body weight. Black line-vehicle treatment, red line, B2M-KO-TSC treatment. Mean ± standard deviation, *n* = 4–7. **(B)** Heart rate before SM-challenge and on post-SM-challenge days 1–28 is reported as beats per minute. Black line-vehicle treatment, red line, B2M-KO-TSC treatment. Mean ± standard deviation, *n* = 4–7. **(C)** Oxygen saturation before SM-challenge and on post-SM-challenge days 1–28 is reported as the percent of baseline. Black line-vehicle treatment, red line, B2M-KO-TSC treatment. Mean ± standard deviation, *n* = 4–7. **(D)** Wet lung weight on post-SM-challenge (PC) days 7, 14 and 28 in vehicle (black bars) and B2M-KO-TSC (red bars) treated rats. Mean ± standard deviation, *n* = 4–7. **(E)** Total lung protein in bronchoalveolar lavage on PC days 7, 14 and 28 in vehicle (black bars) and B2M-KO-TSC (red bars) treated rats. Mean ± standard deviation, *n* = 4–8. **(F)** TGFβ1 protein concentration in bronchoalveolar lavage on PC days 7, 14 and 28 in vehicle (black bars) and B2M-KO-TSC (red bars) treated rats. Mean ± standard deviation, *n* = 3–8. **(G)** Frequency of macrophages in bronchoalveolar lavage on PC days 7, 14 and 28 in vehicle (black bars) and B2M-KO-TSC (red bars) treated rats. Mean ± standard deviation, *n* = 2–8. **(H)** Frequency of neutrophils in bronchoalveolar lavage on PC days 7, 14 and 28 in vehicle (black bars) and B2M-KO-TSC (red bars) treated rats. Mean ± standard deviation, *n* = 2–8. **(I)** Frequency of eosinophils in bronchoalveolar lavage on PC days 7, 14 and 28 in vehicle (black bars) and B2M-KO-TSC (red bars) treated rats. Mean ± standard deviation, *n* = 2–8.

Since body weight was a quantitative indicator of epithelial injury/repair in the NA transplantation model, body weight was used to follow injury in SM challenged rats (Figure 5A). Body weight decreased to ∼85% of the initial value by day 3 and returned to normal by day 10. No differences were observed for vehicle and rat B2M-KO-TSC treated rats. Similarly, heart rate (Figure 5B), oxygen saturation (Figure 5C), wet lung weight (Figure 5D), and total lung protein in the lung lavage (Figure 5E) did not vary by treatment. TGFβ1, a proinflammatory marker identified in SM-challenged rats (McGraw et al., 2018), was decreased in B2M-KO-TSC treated rats on day 7 day but did not vary at later timepoints (Figure 5F). Other biomarkers of SM exposure, MMP9, PDGFβ, and PIA1 did not vary between the treatment groups (data not shown). Similarly, macrophage, neutrophil, and eosinophil numbers did not differ between the two groups (Figures 5G–I). Very few lymphocytes or basophils were detected and did not vary by treatment (data not shown). Specifically, lymphocytes were 0.3667 ± 1.217% (range 0–6%, with of 26 of 30 rats having zero lymphocytes) cells in the bronchoalveolar lavage. These data indicate that there were no treatment dependent differences in the frequency of any leukocyte subtype including lymphocytes.

## Discussion

Cas9 gene editing has been introduced into the clinic and promising outcomes have been reported (Gillmore et al., 2021). In the present study, gene editing technology allowed production of B2M-KO-TSC which were MHC-1 negative. This innovation was the initial step toward development of an allogeneic TSC therapy which could be used to treat various lung diseases including the long-term effects of SM exposure.

Since B2M-KO-TSC were designed to avoid immune detection, off-target gene edits were of significant concern. Whole genome sequencing and Churchill analysis of human B2M-KO-TSC demonstrated that introduction of Cas9/RNP molecules by electroporation resulted in few off-target modifications and that these unintended alterations were significantly less frequent than those reported for stable lentiviral transduction of the gene editing reagents (Kim et al., 2014; Vakulskas and Behlke, 2019). The low number of off-target edits was likely due to transient expression of Cas9. Demonstration that human B2M-KO-TSC self-renewed and underwent multilineage differentiation reinforced the conclusion that edited TSC retained critical functional characteristics and that off-target effects were benign.

Our previous TSC transplantation study reported that human and mouse TSC engrafted the tracheal epithelium of NSG mice but not the intrapulmonary airways (Ghosh et al., 2017). In contrast, we now report engraftment of both compartments by rat B2M-KO-TSC. A technical explanation for this difference is unlikely since cell dose, cell delivery route, treatment timing, and host strain did not very between the two studies. However, we do note that the female host mice in the present study lost more body weight than in the previous study and required treatment with saline on days 2–4. Further, club and ciliated cell frequency was less than normal on post-NA challenge day 30, a finding that indicates that the mice in the present study were more severely injured than those in our previous report. These data indicate that severe injury and a prolonged recovery period enhance epithelial engraftment.

Another possible explanation for bronchiolar engraftment is that rat TSC are more capable of populating the bronchiolar epithelium than human and mouse TSC. While it was anticipated that rat TSC would home to the trachea, which is the location of rat and mouse TSC, it is possible that rat TSC express adhesion proteins which allow them to adhere to bronchiolar cells and/or extracellular matrix. A comparative analysis of human, mouse, and rat TSC may reveal the identity of these molecules and lead to improved efficacy of cellular therapies.

Finally, it is possible that rat TSC engrafted the trachea initially and then migrated into the intrapulmonary airways. This explanation is supported by recent studies showing that airway basal cells, including TSC, migrate long distances in response to severe injury (Kumar et al., 2011; Lynch et al., 2018; Jr et al., 2021). If future studies support this mechanism, it is possible that TSC delivery to the tracheal region will lead to effective repopulation of the entire airway epithelium and simplify the cell delivery component of cell therapy approaches.

Our preclinical assessment of allogeneic B2M-KO-TSC therapy in rats is, to the best of our knowledge, the first of its kind. Our analysis of rat B2M-KO-TSC treated/SM challenged rats indicates that the cell therapy did not elicit any adverse events across the timeline of the study. Specifically, cell treatment did not exacerbate SM-induced deaths or changes in respiratory injury parameters, respiratory function, lung inflammation, or cardiac function. However, we were not able to demonstrate efficacy of the proposed MCM. There are several potential explanations for this finding.

First, it is possible that the SM-induced injury was not sufficient to permit engraftment of B2M-KO-TSC. Relative to our NA engraftment study, the body weight decrease observed in SM-challenged rats was lower and short-lived. However, histological analysis of lung tissue demonstrated that airway epithelial damage was severe and suggested that SM-induced epithelial injury cleared the stem cell niche. Second, it is possible that the rat B2M-KO-TSC did not traffic from vasculature into the epithelium. Although our previous study indicated that TSC engraftment required instillation into the airway lumen, a report that SM challenge caused vascular leak (Veress et al., 2013) led us to test intravascular administration of rat B2M-KO-TSC. In the present study, vascular leak was detected in a subset of animals, but no animals developed plastic bronchitis. Consequently, engraftment may have been limited by the route of delivery rather than the competence of the test cells. Successful engraftment of rat B2M-KO-TSC that were delivered by intratracheal instillation into NA injured mice indicates that these cells could engraft the trachea and lung and suggests that intratracheal instillation should be tested as a delivery route in future studies. Finally, it is possible that lentiviral transduction led to detection of the rat B2M-KO-TSC by the intact immune system of SM challenged rats. While we do not have direct evidence of this mechanism, viral antigens are detected by immune cells including natural killer cells (NKC) and rapid B2M-KO-TSC killing by NKC could explain our failure to detect transplanted cells in immunocompetent rats. In contrast with the SM-challenge study, the NSG mice used in the NA engraftment study lack NKC and this deficiency could have protected the virally transduced cells. Pending future identification of a NKC-dependent mechanism, previously reported methods (Koga et al., 2020) could be used to force expression of CD47, a natural killer cell inhibitory ligand, and allow rat B2M-KO-TSC to avoid detection by NKC.

## Data Availability

The datasets presented in this article are not readily available because we obtained consent to conduct the cell biological studies, but we did not obtain explicit consent to share genomic data or information. Requests to access the datasets should be directed to the corresponding author.
