# Supplementary figures and images for: Assessment of Beta-2 Microglobulin Gene Edited Airway Epithelial Stem Cells as a treatment for Sulfur Mustard Inhalation
Source: Front Genome Ed. 2022 Feb 7;4:781531. doi: 10.3389/fgeed.2022.781531 (PMC8859869; doi:10.3389/fgeed.2022.781531)

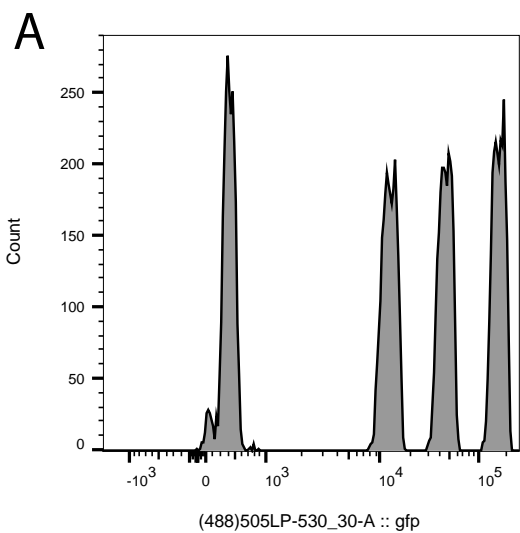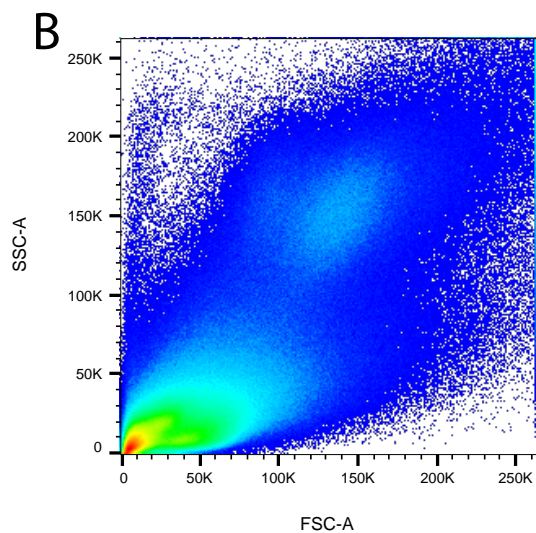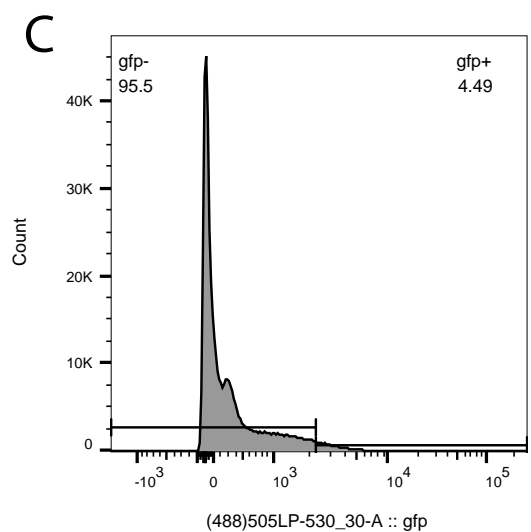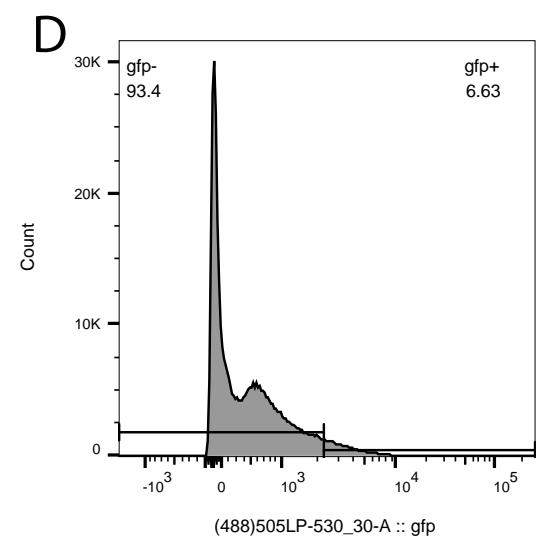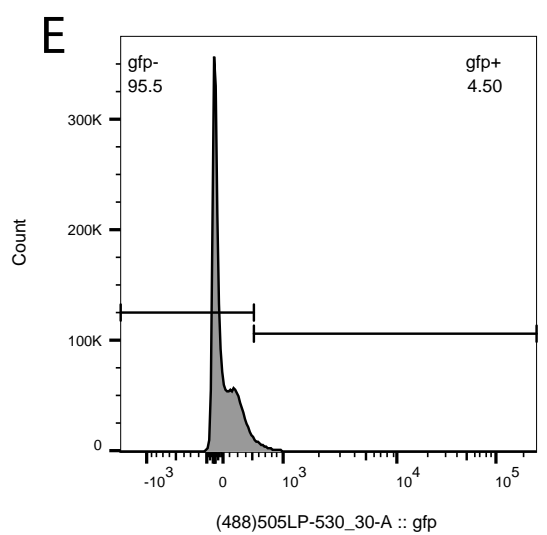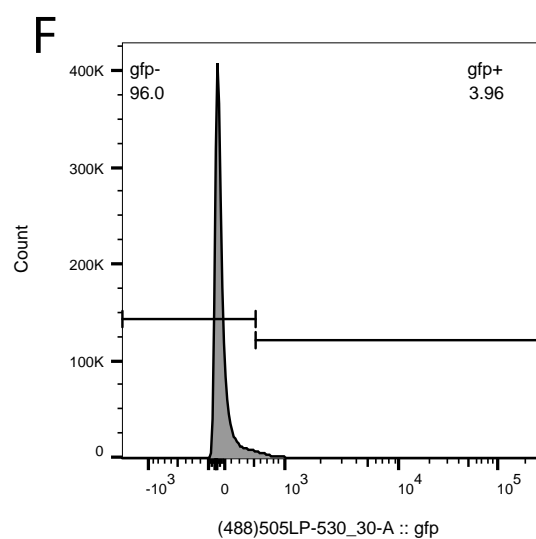

Supplement: Supplementary file 1 [file DataSheet2.PDF]

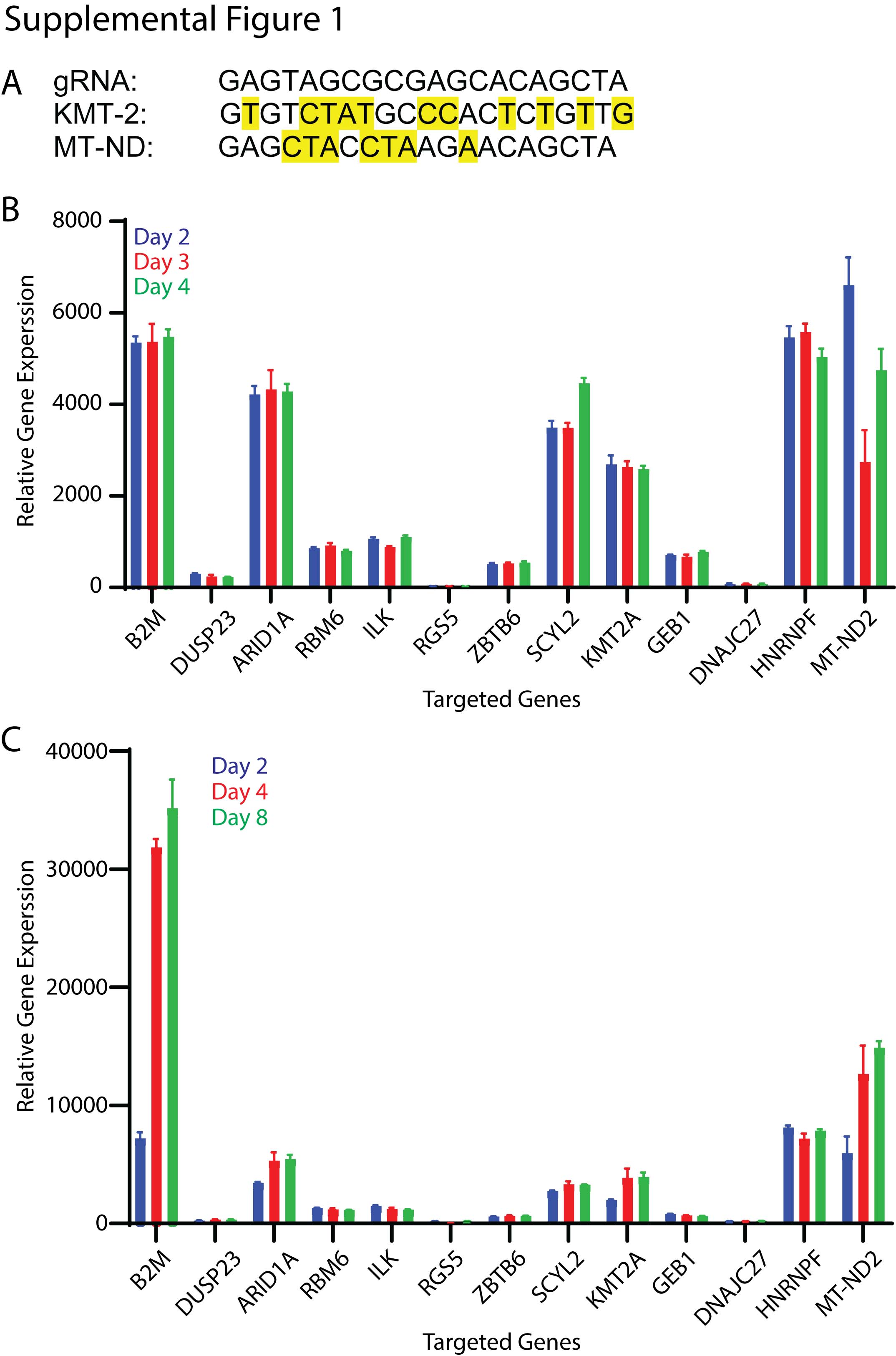

Supplement: Supplementary file 3 [file Image1.JPEG]

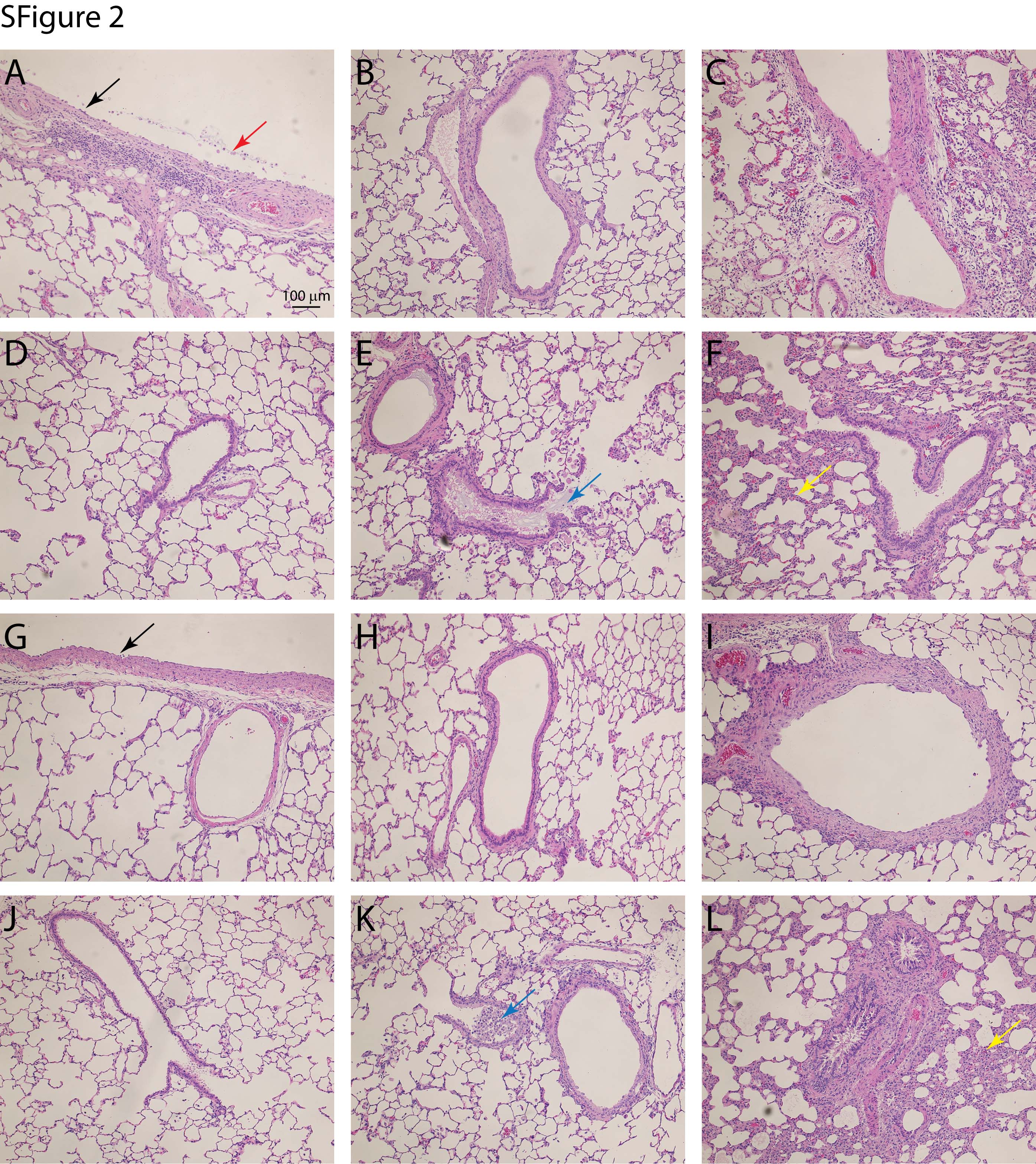

Supplement: Supplementary file 4 [file Image2.JPEG]

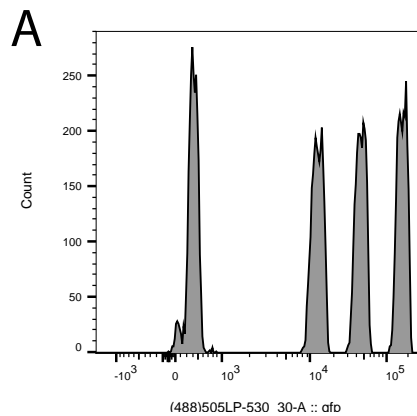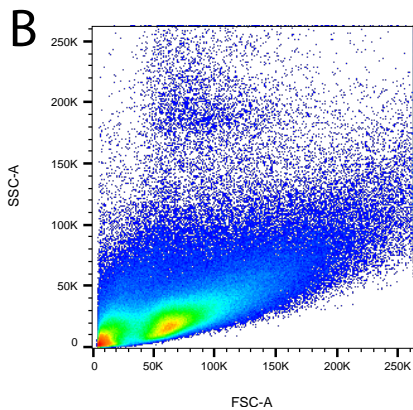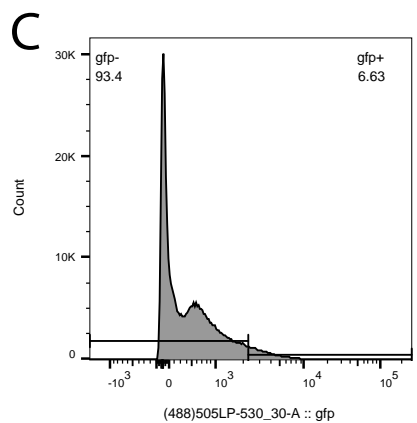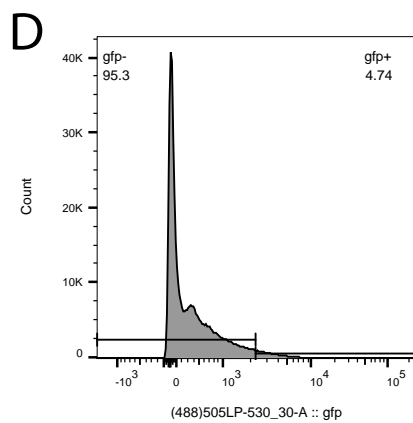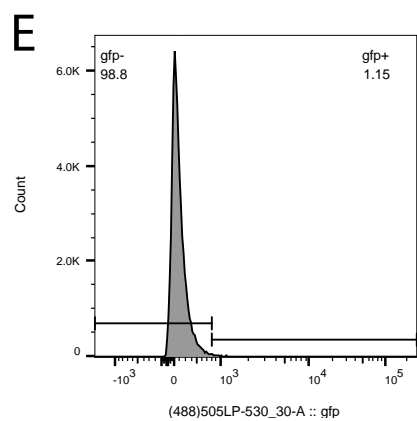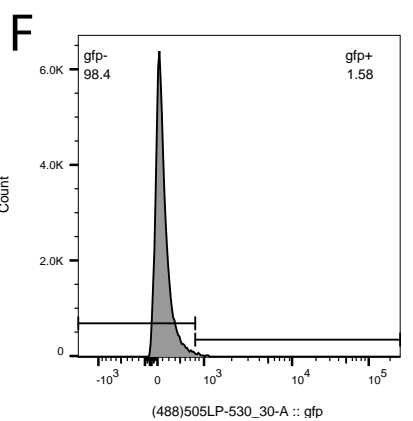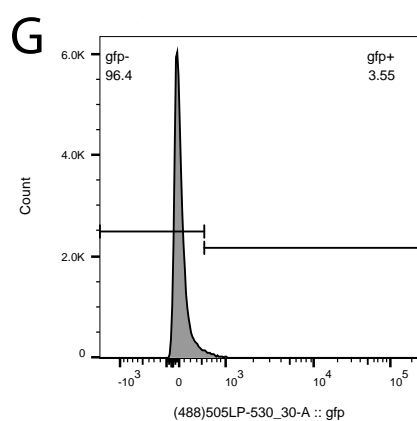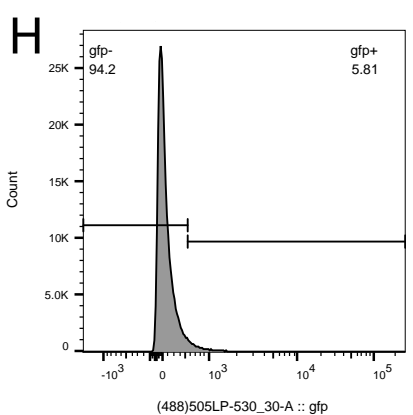

Supplement: Supplementary file 5 [file DataSheet1.PDF]
