# Supplementary material for: Assessment of Beta-2 Microglobulin Gene Edited Airway Epithelial Stem Cells as a treatment for Sulfur Mustard Inhalation
Source: Front Genome Ed. 2022 Feb 7;4:781531. doi: 10.3389/fgeed.2022.781531 (PMC8859869; doi:10.3389/fgeed.2022.781531)
Supplement: Supplementary file 2 [file Table1.docx]

| STable 1: Frequency of EGFP-Positive Cells | | | | | | | | |
| --- | --- | --- | --- | --- | --- | --- | --- | --- |
| Tissue | Time | Vehicle | | | Cells | | | p-value |
|  |  | Mean | SD | n | Mean | SD | n |  |
| Trachea | day 7 | 7.45 | 2.98 | 5.00 | 4.89 | 2.20 | 8.00 | 0.14 |
|  | day 14 | 1.92 | 1.29 | 5.00 | 1.75 | 0.83 | 4.00 | 0.82 |
|  | day 21 | 4.73 | 5.08 | 4.00 | 9.96 | 5.86 | 2.00 | 0.40 |
| Lung | day 7 | 3.77 | 3.22 | 7.00 | 2.77 | 2.87 | 13.00 | 0.51 |
|  | day 14 | Not done | | | Not done | | |  |
|  | day 21 | 4.73 | 2.07 | 4.00 | 4.84 | 1.73 | 5.00 | 0.93 |
